# SARS-CoV-2 Nucleocapsid Protein Is a Potential Therapeutic Target for Anticoronavirus Drug Discovery

**DOI:** 10.1128/spectrum.01186-23

**Published:** 2023-05-18

**Authors:** Austin Royster, Songyang Ren, Yutian Ma, Melissa Pintado, Eunice Kahng, Sean Rowan, Sheema Mir, Mohammad Mir

**Affiliations:** a Western University of Health Sciences, Pomona, California, USA; Institute of Microbiology Chinese Academy of Sciences

**Keywords:** coronavirus, RNA virus, antiviral agents, nucleocapsid protein, virus replication

## Abstract

SARS-CoV-2, the etiologic agent of the COVID-19 pandemic, is a highly contagious positive-sense RNA virus. Its explosive community spread and the emergence of new mutant strains have created palpable anxiety even in vaccinated people. The lack of effective anticoronavirus therapeutics continues to be a major global health concern, especially due to the high evolution rate of SARS-CoV-2. The nucleocapsid protein (N protein) of SARS-CoV-2 is highly conserved and involved in diverse processes of the virus replication cycle. Despite its critical role in coronavirus replication, N protein remains an unexplored target for anticoronavirus drug discovery. Here, we demonstrate that a novel compound, K31, binds to the N protein of SARS-CoV-2 and noncompetitively inhibits its binding to the 5′ terminus of the viral genomic RNA. K31 is well tolerated by SARS-CoV-2-permissive Caco2 cells. Our results show that K31 inhibited SARS-CoV-2 replication in Caco2 cells with a selective index of ~58. These observations suggest that SARS-CoV-2 N protein is a druggable target for anticoronavirus drug discovery. K31 holds promise for further development as an anticoronavirus therapeutic.

**IMPORTANCE** The lack of potent antiviral drugs for SARS-CoV-2 is a serious global health concern, especially with the explosive spread of the COVID-19 pandemic worldwide and the constant emergence of new mutant strains with improved human-to-human transmission. Although an effective coronavirus vaccine appears promising, the lengthy vaccine development processes in general and the emergence of new mutant viral strains with a potential to evade the vaccine always remain a serious concern. The antiviral drugs targeted to the highly conserved targets of viral or host origin remain the most viable and timely approach, easily accessible to the general population, in combating any new viral illness. The majority of anticoronavirus drug development efforts have focused on spike protein, envelope protein, 3CL^pro^, and M^pro^. Our results show that virus-encoded N protein is a novel therapeutic target for anticoronavirus drug discovery. Due to its high conservation, the anti-N protein inhibitors will likely have broad-spectrum anticoronavirus activity.

## OBSERVATION

**FIG 1:**
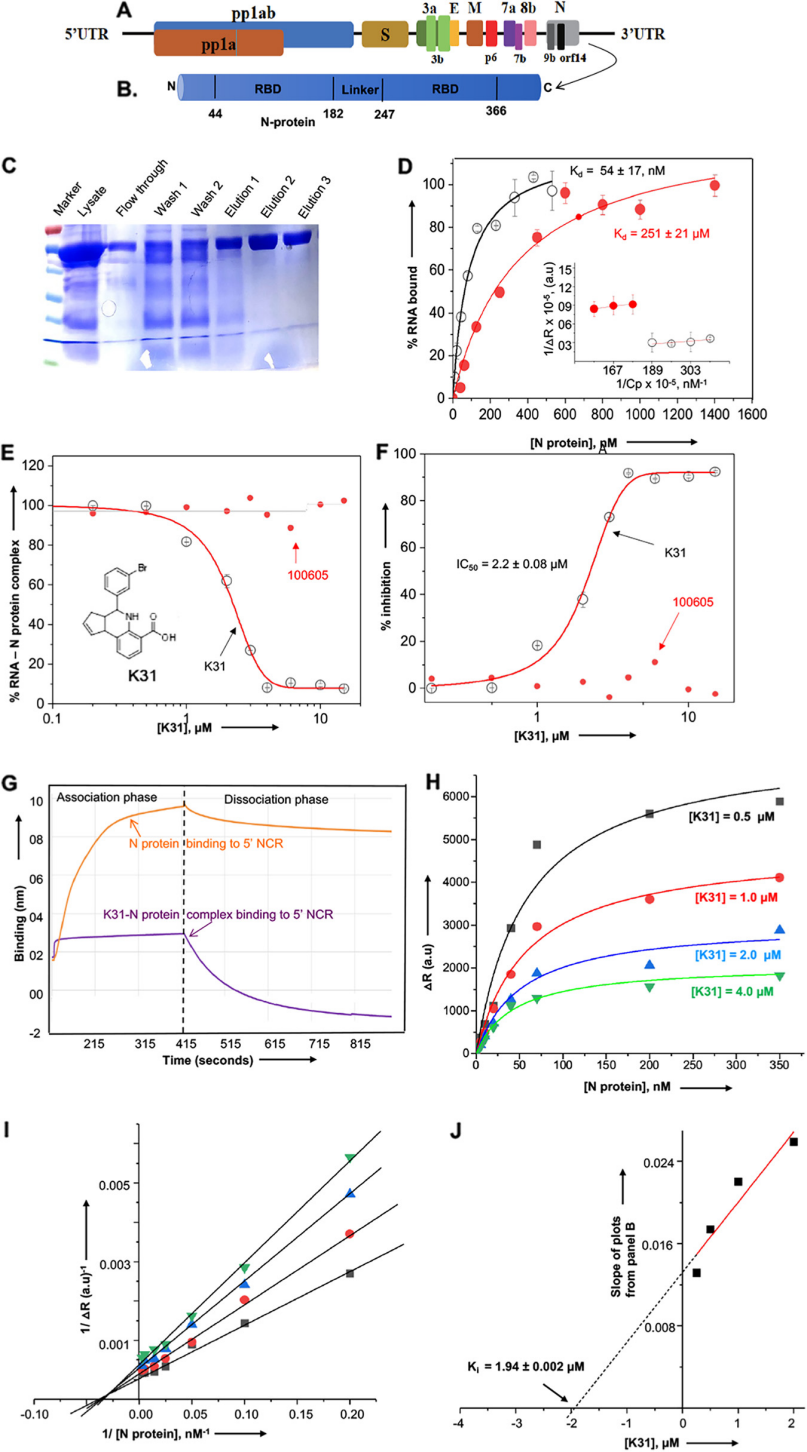
K31 binds to the SARS-CoV-2 N protein and inhibits interaction with the 5′ terminus of viral genomic RNA. (A) Schematic diagram of the genome organization of SARS-CoV-2 (IVDC-HB-01/2019 HB01 strain) (1). (B) Schematic diagram of domain representation of SARS-CoV-2 N protein (33, 34). RBD, RNA binding domain. (C) Purification of C-terminally His-tagged SARS-CoV-2 N protein using Ni-NTA chromatography. (D) Binding profiles for the interaction of SARS-CoV-2 N protein with 5′ NCR sequence (open circles) and 5′ UTR of hantaviral mRNA (filled red circles) using a filter binding assay. The inset shows a double reciprocal plot. For details about the filter binding assay and generation of binding profiles, please see references 21, 27, and 28. a.u., arbitrary units. (E) Inhibition profiles showing the percentage of radiolabeled 5′ NCR-N protein complex retained on the filter at increasing input concentrations of K31 (open circles) and inactive compound 100605 (filled red circles), previously identified in a high-throughput screen (16, 17). (F) The data from panel E were used to calculate percent inhibition at each input concentration of K31 or compound 100605. The data points were fitted to a dose-response curve using Origin 6.0 Pro for the calculation of EC_50_ values. (G) Biolayer interferometry showing the association and dissociation kinetics for the binding of purified N protein to the immobilized biotinylated 5′ NCR in the absence (orange) and presence (purple) of K31. (H) Binding profiles for N protein-5′ NCR interaction at four different K31 concentrations, as shown. (I) The Lineweaver-Burk plots were generated using the data from panel H. (J) The secondary plot was generated by plotting the slopes of Lineweaver-Burk plots from panel I versus input K31 concentration. The data points were fitted to a straight line.

The SARS-CoV-2 genome, having a close evolutionary association with the SARS-like bat coronaviruses, has 14 open reading frames (Fig. 1A) encoding 27 proteins, including the nucleocapsid protein (N protein) (1). The coronavirus N protein is highly conserved and plays diverse roles in the virus replication cycle, such as encapsidation of the viral genomic RNA, transcription and replication of the viral genome in conjunction with the replication complex (2), viral assembly (3), virus budding (4), cell cycle regulation (5), immune system interference (6), and host translation shutoff (7). The N protein has a highly conserved structural architecture, composed of N-terminal and C-terminal RNA binding domains separated by a disordered linker region (Fig. 1B). Since the emergence of SARS-CoV-2 in the city of Wuhan in China, several SARS-CoV-2 variants, such as Alpha (United Kingdom), Beta (South Africa), Gamma (Brazil), Delta (India), Epsilon (California), and Omicron (South Africa), emerged in a time frame of ~2 years (2020 to 2021). Although these variants gained fitness due to mutations in the spike protein, the N protein sequence alignments of the parent Wuhan strain (GenBank accession no. UHN41071.1), the Alpha strain (GenBank no. OQ847681.1), the Beta strain (GenBank no. QRN78355.1), the Gamma strain (GenBank no. QRX39433.1), the Delta strain (GenBank no. QUD52772.1), the Epsilon strain (GenBank no. QQM19149.1), and the Omicron strain BA.4 (GenBank no. UZG29440.1) revealed that their N protein was >98% conserved. The two RNA binding domains (amino acids 44 to 182 and amino acids 247 to 366) of N protein are 100% conserved among all these reported SARS-CoV-2 variants. The majority of the mutations, including three deletions in the Omicron variant, were reported in the first 30 amino acids of the N protein, away from the N-terminal RNA binding domain. Being involved in numerous processes of the coronavirus life cycle, the highly conserved and multifunctional N protein represents a novel target for therapeutic intervention in coronavirus disease. While the majority of anticoronavirus drug development efforts target RNA-dependent RNA polymerase (RdRp) (8, 9), spike protein (10), envelope protein (11), 3CL^pro^ (12, 13), and M^pro^ (14, 15), the N protein as an important therapeutic target has not been much explored. Using a high-throughput screening approach (16, 17), we previously identified a novel molecule [4-(3-bromophenyl)-3a,4,5,9b-tetrahydro-3H-cyclopenta[c]quinoline-6-carboxylic acid], referred to here as K31 (Fig. 1E, inset), that targets the hantavirus nucleocapsid protein and inhibits hantavirus replication in cells with a selective index (SI) of >18 (18–20). We demonstrated that K31 binds to the hantavirus nucleocapsid protein and inhibits its interaction with the viral mRNA 5′ untranslated region (5′ UTR) (16, 17). K31-mediated dissociation of the N protein-5′ UTR complex inhibited hantavirus replication in cells with a selective index of ~18.0 (16, 17). Here, we demonstrate that K31 binds to the nucleocapsid protein of SARS-CoV-2 and inhibits its replication in cell culture with a high selective index.

The gene encoding the N protein of SARS-CoV-2 (isolate Wuhan-Hu-1, NCBI identifier [ID] NC_045512.2) was cloned between NdeI and BamHI restriction sites in the pet30a(+) plasmid and expressed in Escherichia coli as a C-terminally His-tagged fusion protein. The protein was purified on a nickel-nitrilotriacetic acid (Ni-NTA) column using the native purification method, as previously reported (17). As shown in Fig. 1C, the purified N protein is free of detectable heterologous bacterial proteins and its electrophoretic mobility is consistent with its expected molecular mass of ~45.6 kDa.

To determine whether the purified N protein was biologically active, we studied its interaction with the synthetic 5′ noncoding region (5′ NCR) of the SARS-CoV-2 genomic RNA using a filter binding approach, as previously reported (21, 22). Briefly, the 36 nucleotides of the 5′ NCR were synthesized *in vitro* using T7 RNA polymerase and radiolabeled with [α-^32^P]GTP during synthesis. A fixed concentration of radiolabeled 5′ NCR was incubated with increasing concentrations of N protein at room temperature for 30 to 45 min and filtered through nitrocellulose membranes under vacuum. The percentage of radiolabeled RNA retained on the washed filter at each input concentration of N protein was measured by quantifying the radioactive signal, using a scintillation counter, and plotted versus N protein concentration to generate the binding profile (Fig. 1D). The apparent dissociation constant (*K_d_*) corresponded to the concentration of N protein required to obtain half-saturation in the fitted binding curve. This analysis revealed a *K_d_* value of 54 ± 17 nM for the N protein-5′ NCR interaction, confirming the biological activity of the purified N protein. Similar studies revealed that SARS-CoV-2 N protein bound to the 5′ UTR of hantavirus S-segment mRNA with ~5-fold-weaker affinity (*K_d_* of ~251 nM) (Fig. 1D), demonstrating its specificity for binding to the 5′ NCR of SARS-CoV-2.

We next wanted to determine whether K31 inhibits the N protein-5′ NCR interaction *in vitro*. A complex between N protein and 5′ NCR was formed by incubating a fixed concentration of N protein (300 nM) with a fixed concentration of 5′ NCR (~20,000 cpm), followed by incubation of the resulting complex with increasing concentrations of K31 at room temperature for 30 min and filtration of the mixture through a nitrocellulose filter under vacuum. The amount of N protein-5′ NCR complex retained on the filter at each input concentration of K31 was measured by quantifying the radioactive signal, followed by normalization related to the control lacking the K31. The resulting normalized signal was plotted versus K31 concentration to generate the inhibition plots (Fig. 1E and F). The data points were fitted to a dose-response equation using nonlinear least square analysis as previously reported (17). The IC_50_ value represented the concentration of K31 at which 50% of the N protein-5′ NCR complex was dissociated (17). As shown in Fig. 1F, K31 inhibited the N protein-5′ NCR interaction with an IC_50_ value of 2.2 ± 0.08 μM. The experiment was repeated with an inactive compound (100605) that inhibits neither hantavirus replication nor the interaction between hantavirus N protein and viral mRNA 5′ UTR, as previously reported (16, 17). This compound did not inhibit the N protein-5′ NCR interaction in this experiment (Fig. 1F).

To further confirm the inhibition of N protein-5′ NCR interaction by K31, we studied the binding of N protein with 5′ NCR in the presence and absence of K31 using biolayer interferometry (BLI), as previously reported (23). Briefly, the 5′ NCR sequence was synthesized *in vitro* by T7 RNA polymerase and biotinylated during synthesis by the addition of biotinylated CTP to the reaction mixture as previously reported (17, 24–29). The biotinylated RNA was purified and immobilized on a high-precision streptavidin biosensor. The association and dissociation kinetics of the purified N protein with the immobilized RNA were examined, and the resulting data were fitted to a 1:1 binding model, using BLItz software. As shown in Fig. 1G, N protein bound to the 5′ NCR with a high on-rate (*K*_ass_ = 6.775 × 10^4^ M^−1^ s^−1^) and a low off-rate (*K*_dis_ = 3.66610^−3^ s^−1^), generating a dissociation constant, *K_d_*, of 44 ± 7.7 nM (*K_d_* = *K*_dis_/*K*_ass_). A similar *K_d_* value was observed by the filter binding approach (Fig. 1D). However, the binding of N protein with biotinylated 5′ NCR in the presence of 20 μM K31 showed a negligible binding signal (compare the two kinetic profiles in Fig. 1G). Due to poor binding, the *K_d_* values could not be calculated. These experiments clearly demonstrate that K31 inhibits the binding of SARS-CoV-2 N protein to the viral RNA (vRNA) 5′ terminus.

**FIG 2:**
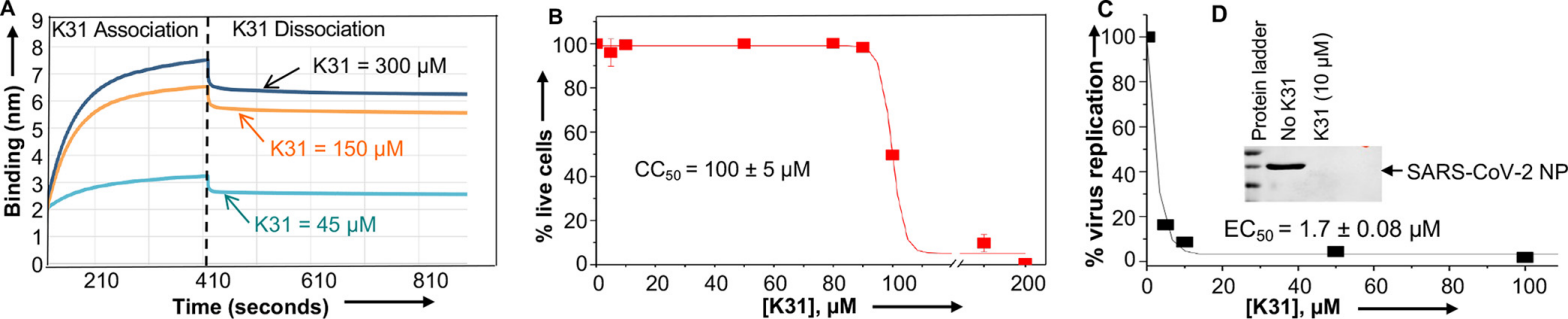
K31 binds to SARS-CoV-2 N protein and inhibits virus replication in cells. (A) Biolayer interferometry showing the association and dissociation kinetics for the binding of K31 with C-terminally His-tagged N protein, immobilized on a Ni-NTA biosensor. The experiment was repeated at three different concentrations of K31 as shown. (B) Cytotoxicity of K31 on Caco2 cells. Caco2 cells in 96-well plates were treated with increasing concentrations of K31 for 3 days and examined for cytotoxicity using a CellTiter-Glo luminescent assay (see reference 17 for details). (C) Inhibition profile showing the percentage of SARS-CoV-2 replication in Caco2 cells at increasing concentrations of K31. SARS-CoV-2 replication was determined by quantification of viral genomic RNA using real-time PCR. (D) Western blot analysis showing N protein levels in SARS-CoV-2-infected Caco2 cells in the absence or presence of 10 μM K31.

To define the mechanism of inhibition, N protein-5′ NCR complexes were formed by incubating a fixed concentration of radiolabeled 5′ NCR (~6,000 cpm) with increasing concentrations of N protein ranging from 0 to 350 nM at room temperature for 30 min. The resulting complexes were further incubated with K31 at four different concentrations (0.5 to 4.0 μM) at room temperature for an additional 30 min, followed by filtration of the mixture through a nitrocellulose filter. The radioactive signal retained on the washed filters was subtracted from the background signal at an N protein concentration of zero to calculate Δ*R*. The resulting Δ*R* values were plotted versus input N protein concentration to generate four binding profiles corresponding to four K31 concentrations (Fig. 1H). The Lineweaver-Burk plots (Fig. 1I) were generated by plotting 1/Δ*R* versus 1/[N protein], and the data points were fitted to a straight line. The characteristic nature of Lineweaver-Burk plots (Fig. 1I) demonstrates that K31 inhibited the N protein-5′ NCR interaction in a noncompetitive manner (30–32). The slopes from four Lineweaver-Burk plots corresponding to four K31 concentrations were plotted versus input K31 concentrations and fitted to a straight line (Fig. 1J). The *x* intercept on the secondary plot (Fig. 1J) demonstrated a *K_i_* (the dissociation constant for the N protein-K31 interaction) of 1.94 ± 0.002 μM. To further confirm that the observed inhibition of the N protein-5′ NCR interaction was due to the binding of K31 with the N protein, we used biolayer interferometry to monitor the potential binding of K31 to the N protein, as mentioned above. As shown in Fig. 2A, K31 bound to the N protein with a high on-rate (*K*_ass_ = 1.071 × 10^2^ M^−1^ s^−1^) and a low-off rate (*K*_dis_ = 2.19210^−4^ s^−1^), generating a dissociation constant (*K_d_*) of ~2 μM (*K_d_* = *K*_dis_/*K*_ass_). A similar binding affinity (*K_i_* of ~1.94 ± 0.002 μM) was observed with Lineweaver-Burk plots (Fig. 1J).

Taken together, the results from two independent experimental approaches (filter binding and biolayer interferometry) clearly demonstrate that purified SARS-CoV-2 N protein binds to the 5′ NCR of viral genomic RNA with high affinity (*K_d_* of ~54 ± 17 nM) (Fig. 1D). The inhibitor K31 binds to the N protein (*K_i_* of ~2 μM) (Fig. 1J and Fig. 2A) and noncompetitively inhibits its binding to the 5′ terminus of viral genomic RNA (IC_50_ = 2.2 ± 0.08 μM) (Fig. 1F). Thus, it was necessary to test its antiviral activity against SARS-CoV-2 in cell culture. Before testing the antiviral activity, we examined the cytotoxicity of K31 in SARS-CoV-2-permissive Caco2 cells using a well-established CellTiter-Glo luminescent assay, as previously reported (17). As shown in Fig. 2B, the CC_50_ (the concentration of K31 at which 50% cell death occurred) was 115 μM, suggesting a considerable tolerance of K31 by this cell line. Similar tolerance was previously reported in multiple mammalian cell lines (17). K31 was further assayed for antiviral activity. Briefly, Caco2 cells seeded in six-well plates were infected with SARS-CoV-2 (strain USA-WA1/2020, GenBank no. MT246667.1) at a multiplicity of infection (MOI) of 0.1 and treated with either vehicle (dimethyl sulfoxide [DMSO]) or increasing concentrations of K31. This experiment was carried out at the Keck School of Medicine, University of Southern California. Cells were washed 1 h postinfection and incubated for an additional 3 days with fresh medium containing K31. Virus replication was monitored by quantitative estimation of viral genomic RNA by real-time PCR. The inhibition plot (Fig. 2C) was used for the calculation of EC_50_, the concentration of K31 at which 50% virus replication was inhibited. This analysis revealed that K31 inhibited SARS-CoV-2 replication in cell culture with an EC_50_ of 1.7 ± 0.2 μM and a selective index (SI) of ~58 (SI = CC_50_/EC_50_). Western blot analysis revealed undetectable levels of N protein in SARS-CoV-2-infected Caco2 cells treated with 10 μM K31 for 3 days postinfection (Fig. 2D). Taken together, these observations suggest that SARS-CoV-2 N protein is a druggable target for anticoronavirus drug discovery, and K31 holds promise for further development as an anticoronavirus therapeutic. In future, a cocrystal structure of the N protein-K31 complex will be determined, which will not only identify the K31 binding site on N protein but will also provide crucial insights for molecular contacts between K31 and N protein, which will be instrumental for further modification of K31 to generate new derivatives that might have high target binding affinity and improved antiviral efficacy. During the COVID-19 pandemic, the sequence of N protein remained >98% conserved among different SARS-CoV-2 variants, suggesting that emergence of K31 drug-resistant mutants due to K31-induced selection pressure is less likely.
